# The Potential Role of Naltrexone in Borderline Personality Disorder

**Published:** 2017-04

**Authors:** Azadeh Moghaddas, Mehrnoush Dianatkhah, Saba Ghaffari, Padideh Ghaeli

**Affiliations:** 1 Department of Clinical Pharmacy, Faculty of Pharmacy, Isfahan University of Medical Sciences, Isfahan, Iran.; 2 Department of Clinical Pharmacy, Faculty of Pharmacy, Tehran University of Medical Sciences, Tehran, Iran.; 3 Department of Clinical Pharmacy, Faculty of Pharmacy, Tabriz University of Medical Sciences, Tabriz, Iran.; 4 Psychiatry and Psychology Research Center, Roozbeh Hospital and Faculty of Pharmacy, Tehran University of Medical Sciences, Tehran, Iran.

**Keywords:** *Borderline Personality Disorder*, *Naltrexone*, *Self-Injurious Behavior*, *Dissociative Symptoms*, *Psychiatry and Psychology Category*

## Abstract

**Objective:** Management of borderline personality disorder (BPD) is a difficult challenge due to the complex features of this disorder. This article reviews the use of naltrexone in the treatment of BPD.

**Method: **Published articles and clinical trials were searched in Google Scholar, MedLine, ELSEVIER, and Cochrane database of systematic reviews abstracts in English language between 1990 and 2017.

**Results: **Naltrexone (NTX), a nonspecific competitive opiate antagonist, has been noted to be helpful in controlling self-injurious behavior (SIB) and dissociative symptoms in patients with BPD.

**Conclusion: **Further studies should be conducted on the effects of naltrexone to confirm the role of this medication in the treatment of BPD.

Borderline Personality Disorder (BPD) is a cluster-B personality disorder that is characterized by marked impulsivity as well as instability in affect regulation, interpersonal relationships, and self-image (1). In a recent epidemiologic survey conducted in the US, the lifetime prevalence of BPD was estimated to be about 5.9% (2). Mood changes and impulsivity are common symptoms observed in both BPD and bipolar disorders. Patients with BPD usually are full of anger and have violent outbursts. Recurring mood changes, usually lasting less than a few hours, repeated suicidal behavior, threats, or gestures, or self-mutilating acts (such as burning or cutting) are listed under BPD criteria in DSM-5. The risk for attempting suicide in the future has been reported to be increased in patients with BPD (3).

Anxiety, eating disorders, and substance abuse are known as highly observed comorbidities with BPD (5).

Managing BPD is challenging and is focused on treating the BPD symptoms. Standard management of BPD consists of psychotherapy as the backbone of the treatment and pharmacotherapy as an adjunct strategy (5). Medications such as serotonin reuptake inhibitors (SSRIs), antipsychotics, mood stabilizers, and anxiolytics are reported to be helpful in the treatment of BPD symptoms.

***Endogenous Opioid System***

The underlying neurobiological mechanism of BPD remains unclear. Alternation in the sensitivity of opioid receptors or the availability of endogenous opiates has been among the proposed neurobiological mechanisms of this disorder. Prossin et al. has measured the availability of Mu-opioid receptor in vivo by radiotracer during sustained sadness of female patients with BPD.

The distribution of involved Mu-opioid receptor exists in regions linked to emotional processing, decision- making, and pain and neuroendocrine regulating (4).

The negative effects of opioid receptor agonists on BPD complications have also been noted in the literature. For example, in a case report by Thüraufet al. (2000), it was cited that consumption of potent analgesics in BPD may increase SIB that may result in severe and life-threatening injuries (6). The endogenous opioid system has been suggested to be responsible for certain clinical features such as stress related analgesia, dissociative behavior, and self-mutilating activity associated with BPD (7). Patients with BPD may unconsciously attempt to stimulate endogenous opioid and the dopaminergic reward systems by acting self-destructively like consuming substances that target opioid receptors without considerations of the consequences (8).

The exact mechanism of endogenous opioid system is not clear, but the role is evitable. One theory suggests that there are functional disturbances in the endogenous opioid system in BPD patients. Due to the disturbances and excessive basal activity in this system, self-injury commitment is not painful for BPD patients. Another theory named “addiction hypothesis” suggests that self-injury and stereotypic behavior may stimulate production and release of endorphins (9).

***Naltrexone***

Naltrexone (NTX) is a nonspecific competitive opiate antagonist that highly binds to the mu- receptor, followed by kappa and delta receptors, hindering the activity of opiates. Because endogenous opioids are involved in modulating the expression of opiates and alcohol’s reinforcing effects, NTX is clinically useful in the treatment of opiate and alcohol abuse (10). In 1984, Food and Drug Administration (FDA) approved NTX for the treatment of opiate dependence. However, NTX is mainly used in treating opiate dependence as a post withdrawal relapse prevention intervention (11). NTX has been noted to be effective in decreasing the reinforcing effects of exploitable substances and their consumption in opioid and alcohol dependent patients. However, this medication has also been suggested as a potential treatment option in several psychiatric disorders such as obesity, eating disorders, schizophrenia, self-mutilation, and other impulse control disorders (12).

The present study aimed at briefly reviewing the existing evidence on the effects of NTX in managing BPD by focusing on 2 prominent features of BPD: self-injurious behavior (SIB) and the dissociative symptoms. Because to date, no medication has obtained FDA approval for the treatment of BPD, medications like NTX need further investigations to find new ways to manage patients with BPD.

## Materials and Methods

The authors considered searching published articles and clinical trials in Google Scholar, MEDLINE, ELSEVIER, and Cochrane database of systematic reviews abstracts in English language between 1990 and 2017. 

## Results

***Naltrexone and Self-Injurious Behavior in BPD Patients*** ***:***

Self-injurious behavior, defined as self-directed act, represents a chronic and often treatment-refractory condition associated with a variety of psychiatric disorders. SIB seems to be a learned behavior for which underling mechanisms such as positive and negative reinforcement, as well as self-stimulation have been proposed (13).

Interest is growing in developing effective treatments for individuals with SIB due to the frequent hospitalization of these patients. For minor acts, psychotherapeutic treatment modalities including social skills training and behavioral therapies are preferred ways of managing (14). However, for moderate or severe SIB, therapeutic modalities such as behavioral and psychopharmacologic interventions are only helpful to some extent. Different pharmacological medication classes have been studied in SIB treatment with antipsychotics being possibly used more than others (15).

Efficacious response to pharmacotherapy has been suggested to be due to influencing both dopaminergic and endogenous opioid systems. On the other hand, it has been noted that other neurotransmitter systems including serotonin and glutamate may also play a role in perpetuating SIB (16).

Furthermore, reduction of SIB symptoms might occur by blockade of the endogenous opioid system. NTX is noted to be one of the best choices for blocking endorphin release (17).

Opioid antagonists like NTX have been studied in the treatment of SIB in individuals with mental retardation and/or autism who exhibit injurious behaviors such as head banging, biting, or face slapping (18-20). A review article by Modesto-Loweet al. has noted a decrease in SIB among patients after NTX administration (12).

Self-injurious behavior is also one of the pathological characters of BPD which affects regulation and self-care (13).

McGee reported the first case of NTX use in the management of BPD in 1997. This case presented a 28-year-old female with BPD, alcohol dependency, recurrent severe major depression, profound trauma, and neglect. She had a strong urge to cut herself to reduce her tension when under stress at work or in psychotherapy sessions. Fluoxetine was prescribed up to 80 mg per day. Although starting fluoxetine reduced depressed mood and suicide thoughts, no improvement in self-mutilation behavior was observed after 2 years of behavioral management and drug therapy. Addition of NTX (50 mg/day) into drug regime led to diminishing compulsive SIB and alcohol craving as well as decreasing the relapse rate (17).

There has been paucity of data about beneficial effects of NTX on SIB, however, in 2000; Thürauf et al. noted the benefits of this medication in reducing SIB symptoms in a female with BPD, who received NTX as a treatment options to control her SIB symptoms (6).

Griengl et al.reported a 39-year-old Caucasian male patient suffering from recurrent severe SIB associated with dysthymia, who was successfully managed by NTX. In this case, SIB flared with episodic irritability, anger and feeling of guilt and it was performed by injection of inflammatory substances such as urine or nitric acid into skin and muscles. After several pharmacotherapeutic approaches including valproic acid, levomepromazin, sertraline, doxepin, and finally risperidone in appropriate dosages, naltrexone 50 mg/day was added to help treat self-destructive behavior. The intervention resulted in impressive cessation of SIB that remained during the 32-week follow-up (21).

Hence, activation of the endogenous opioid system and/or the serotonin system has been suggested to explain analgesia and mood elevation in self-mutilating BPD patients. Treatment with NTX decreased SIB in BPD like other conditions complicated with SIB. It seems that exogenous opioid abolished the mental improvements following self-injury, leading to an increase in SIB because an opioid antagonist like NTX may inhibit aggravation of psychopathology symptom in BPD and has beneficial effects in SIB among this group of patients (17).

***Naltrexone and Dissociative Symptoms in Patients with Borderline Personality Disorder*** ***:***

An important diagnostic criterion in BPD includes dissociative symptoms, which typically worsen under acute stress conditions (22).

Dissociation symptoms include derealization, depersonalization, analgesia, and altered perceptions in several sensory modalities as well as pain sensitivity reduction (23).

These symptoms are a burden and may lead to subsequent complications such as nonsuicidal self-injuries and poor response to behavior therapies in BPD patients. 

Psychotherapy has been the preferred approach for treating dissociative symptoms, but some pharmacotherapeutic options such as fluoxetine and paroxetine have been shown to reduce dissociative symptoms in some BPD (24).

Opioid receptor antagonists such as naloxone and NTX were found to have a promising anti-dissociative effects. For example, Pitman et al. in 1990 assessed the effects of using 2–4mg of naloxone per day in blocking stress-induced analgesia in patients with posttraumatic stress disorder (25).Two small uncontrolled open-label studies reported a reduction in dissociative symptoms in patients with chronic depersonalization (26) and depersonalization disorder (27), respectively.

Bohus et al. in 1999 studied the effects of NTX in patients with BPD and prominent dissociative phenomena including flashbacks in an open-label trial.

They treated 2 groups of females suffering from BPD according to diagnostic criteria of DSM-IV (N = 13, with an overlap of 5 patients between the two groups), who experienced prominent dissociative phenomena including flashbacks. They received NTX 25 to 100 mg QID for at least 2 weeks. Patients were examined by a self-rated questionnaire which measured dissociation, analgesia, tonic immobility, and tension (DAISS) during 7 consecutive days before and during treatment with NTX. In addition, they assessed 9 other patients (with an overlap of 5 patients from the other group) for flashback protocol. Based on DAISS scores, results revealed a highly significant reduction of the duration and the severity of dissociative events and tonic immobility as well as a promising reduction in analgesia during NTX therapy. Most patients experienced a decrease in the mean number of flashbacks per day. The present study had some limitations; it only relied on self-reported phenomena instead of a questionnaire, specially applied for pain or tension reduction assessment, moreover, various doses were used, and most importantly the methodology of the study was based on open-label trial with no placebo for omitting the effects of hospitalization and other treatment protocol drugs. However, the authors proposed conducting a placebo-controlled double-blind study to assess the potential benefit of NTX in a more rigorous way (28). Hence, they examined the effects of naltrexone using a more rigorous design that controlled for major confounders such as spontaneous reduction of dissociation over time and placebo effects. Later, Schmahl et al. 

**Table 1:** The Effect of Psychological Drugs on Symptom of BPD (1)

Medication	Effect
Antipsychotics	Affective dysregulation including inappropriate anger, impulsivity/aggression and interpersonal problems, moderate effects treating cognitive-perceptual symptoms, and small to moderate effects for global functioning
Mood stabilizers	Affective dysregulation including inappropriate anger, impulsivity and aggression, interpersonal problems, and global functioning
Antidepressants	Negligible to small clinical effects for treating the symptoms of dysphoria in the absence of major depression

studied the effects of NTX on dissociative symptoms reduction in BPD patients in a more disciplined design by controlling major confounders such as spontaneous reduction of dissociation over time and placebo effects. A total of 29 drug-naïve BPD patients were assigned into 2 small, placebo-controlled double-blind trials that lasted for 6 weeks (23).

## Discussion

The role of opioid antagonists in controlling the symptom of BPD patients such as dissociative symptoms or SIB is very unclear. There is paucity of data around NTX uses in BPD patients, but indirect evidence from other clinical presentation than BPD might demonstrate that NTX help treat self-injurious thoughts and behaviors (29). For instance, 2 small uncontrolled open-label studies (26, 27) found a reduction in dissociative symptoms in patients primarily suffering from depersonalization. 

Dissociative symptoms may result from a complex interplay of several neurochemical systems. It has been proposed that the effects of NTX on dissociative symptoms may fit current neurobiological models including opioid, serotonergic, and glutamatergic NMDA receptor systems (30).

On the other hand, several hypotheses have been suggested to explain the effects of NTX on SIB. Primarily, it has been declared that SIB may be positively reinforced by the release of endogenous opioids, producing a euphoric effect (31). Although opioid antagonists compete with endogenous opioids at the receptor binding sites, they would not activate them. Receptor blockage by opioid antagonists leads to withholding reinforcing consequences. Thus, it is suggested that frequent stimulation of endogenous opioids system results in physical dependence in SIB (32).Therefore, by preventing endogenous opioids from binding to their receptor sites, NTX withholds the reinforcement to maintain SIB. Another hypothesis suggests that brain opioid activity enhancement generates excessive endogenous opioids, leading to an elevated pain threshold in some individuals (33).

NTX has shown both encouraging and unfavorable effects in the management of SIB and dissociative manner (34, 35,36). Such difference in treatment responses to NTX suggests possible involvement of variety of mechanisms for this medication (32).

## Limitations

The main limitation of this brief review is the fact that a few controlled trials on the use of medications in BPD have been published. To this date, no medication has received FDA-approval for the treatment of BPD. Indeed, many medications like naltrexone are used to only improve distressing symptoms of BPD.

## Conclusion

The role of NTX and other opioids antagonists in managing BPD complication is still controversial and incomplete. However, because no medication has obtained approval in the management of BPD, NTX is worth to be considered for further controlled trials based on some reports on its beneficial effects in reducing SIB in patients with BPD.
